# A localized tracing technique to explore intra-amygdala functional and structural correlates of individual variability in behavioral response

**DOI:** 10.3389/fnmol.2025.1347539

**Published:** 2025-01-22

**Authors:** Allie Lipshutz, Victoria Saltz, Kristin R. Anderson, Alessia Manganaro, Dani Dumitriu

**Affiliations:** ^1^Division of Developmental Neuroscience, Department of Psychiatry, Columbia University, New York, NY, United States; ^2^Division for Child and Adolescent Health, Department of Pediatrics, Columbia University Irving Medical Center, New York, NY, United States; ^3^Zuckerman Institute, Columbia University, New York, NY, United States

**Keywords:** amygdala (amg), retrograde tracing, cFos, microcircuits, stress models of psychopathology

## Abstract

**Introduction:**

The neurobiological basis for individual variability in behavioral responses to stimuli remains poorly understood. Probing the neural substrates that underlie individual variability in stress responses may open the door for preventive approaches that use biological markers to identify at-risk populations. New developments of viral neuronal tracing tools have led to a recent increase in studies on long range circuits and their functional role in stress responses and social behavior. While these studies are necessary to untangle largescale connectivity, most social behaviors are mediated and fine-tuned by local subregional circuitry.

**Methods:**

In order to probe this local, interregional connectivity, we present a new combination of a neuronal tracing system with immediate early gene immunohistochemistry for examining structural and functional connectivity within the same animal. Specifically, we combine a retrograde transsynaptic rabies tracing system with cFos colocalization immediately after an acute stressor to elucidate local structural and stress-activated connectivity within the amygdala complex in female and male mice.

**Results and discussion:**

We show how specific structural and functional connections can predict individual variability along a spectrum of social approach/avoidance following acute social defeat stress. We demonstrate how our robust method can be used to elucidate structural and functional differences in local connectivity that mediate individual variability in behavioral response.

## 1 Introduction

Individual variability in response to analogous stimuli continues to explain a vast discrepancy in pathological outcomes to disease (Zhdanava et al., 2021), yet it remains poorly understood. Identifying biological markers of susceptibility to disease, thus, may open the door for screening protocols and preventative treatment approaches, prior to the onset of stress.

Due to its critical role in the systemic stress response (Tapp et al., 2019; Chakraborty et al., 2020; Almeida et al., 2021), the amygdala complex may be a promising target for identifying pre-existing structural and functional differences that mediate individual variability in the behavioral stress response. Structurally, the amygdala is composed of 13 subnuclei (Sah et al., 2003), intermingled with functionally specific subpopulations of neurons. Each neuronal type has notable extrinsic connectivity with anxiogenic and fear learning-associated regions, including the ventral hippocampus (vHPC) (Xu et al., 2016; Jimenez et al., 2018; Graham et al., 2021), nucleus accumbens (NAc) (Sah et al., 2003; Namburi et al., 2015), prelimbic cortex (PL) (Grossman et al., 2022), and infralimbic cortex (IL) (Senn et al., 2014; Klavir et al., 2017; Bloodgood et al., 2018). Beyond extrinsic connections, these nuclei also have critical intrinsic local connectivity: sensory input enters through the lateral amygdala (LA) which projects to the central lateral amygdala (CeL) and basolateral amygdala (BLA) (Lee et al., 2013). The CeL, specifically, contains multiple inhibitory circuits with opposing functions that serve to mediate a range of responses, and with the BLA, project to the centromedial amygdala (CeM), and finally to the brain stem to initiate a fear response (Haubensak et al., 2010; Ciocchi et al., 2010; Fadok et al., 2017). While long-range connectivity with the amygdala complex in the context of social stress has received significant attention (Anderson and Dumitriu, 2020; Anderson et al., 2021; Grossman et al., 2022), the local intra-amygdala connectivity patterns in this behavioral context remain largely unknown.

Therefore, we sought to develop a method to elucidate local connectivity and activity within the amygdala complex that is both preexisting to any stressor (structural) and/or associated with a behavioral stress response (functional). To identify local connectivity and activity, we implemented the well-validated rabies system as a retrograde transsynaptic tracer (Callaway and Luo, 2015) and a mouse model of acute social defeat stress (ASDS) that induces a spectrum of social approach/avoidance in a social interaction test (SI) 1 h after exposure to acute social stress (Grossman et al., 2022). Immunohistochemical identification of potentially stress-activated neurons was obtained by perfusing the mice immediately after SI and staining for the neural activity dependent protein, cFos (Dragunow and Faull, 1989; Martinez et al., 2002). To determine projection neurons activated by acute stress (Knapska and Maren, 2009; Vialou et al., 2014; Twining et al., 2017; Zhang et al., 2019; Dirven et al., 2022), we quantify the colocalization of G-deleted pseudorabies (RV*dG*) and cFos protein. We use this proof-of-concept experiment to show how connectivity patterns can be mapped onto individual variability in behavioral responses.

## 2 Methods

### 2.1 Animals

Experimental C57BL/6J female and male mice aged 8 weeks were group housed with five mice of the same sex in each cage (*n* = 10). All cages were maintained on a 12-h light/dark cycle (lights on 7:00 am), with food and water ad libitum. All surgical and behavioral experiments were performed during light hours and began when mice were aged 10 weeks. Aggressive retired male breeder CD1 mice from Charles River Laboratories were used for all social defeat experiments and nonaggressive retired male breeder CD1 mice were used for social interaction testing. All experiments were conducted in compliance with National Institutes of Health Guidelines for Care and Use of Experimental Animals and approved by the Institutional Animal Care and Use Committees at Columbia University Irving Medical Center and the New York State Psychiatric Institute.

### 2.2 Stereotaxic surgery

**Figure 1:**
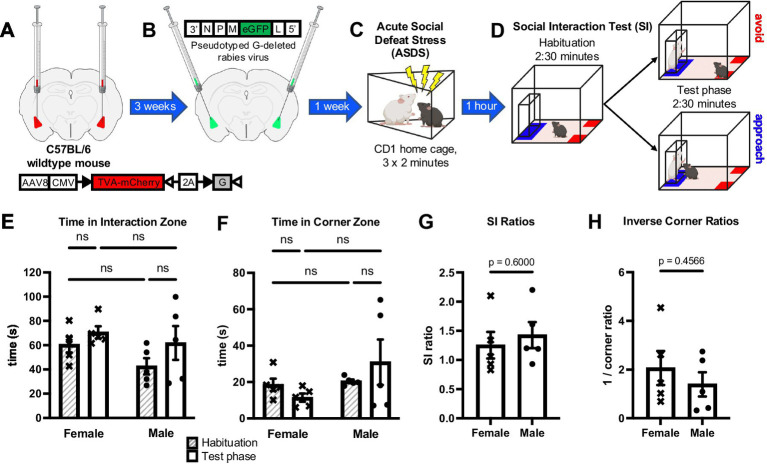
Behavioral responses to acute social defeat stress (ASDS) in female and male mice stereotactically injected for local connectivity tracing in the amygdala complex. **(A)** An mCherry-tagged TVA and G protein-containing viral complex was injected bilaterally into the BLA of female and male C57BL/6 wildtype mice. **(B)** Three weeks later, the same mice were injected with a pseudotyped G-deleted rabies virus into the bilateral BLA at an angle of 11° to promote tracing specificity. **(C)** After a one-week recovery and viral expression period, mice were subjected to ASDS. Each female and male mouse was introduced into the cages of three different territorial aggressive CD1 male mice for a period of 2 min each consecutively. **(D)** A social interaction (SI) test was performed 54 min after ASDS, consisting of 2:30 min of habituation in which the experimental mouse is allowed to explore the arena and 2:30 min of testing in which a nonaggressive CD1 mouse (“target”) was placed in the interaction chamber and the experimental mouse was again allowed to explore. Directly following SI (in all cases 1-h post-ASDS), mice were perfused and brains collected for structural and functional connectivity analyses. **(E)** Female and male mice do not differ statistically in absolute time spent in the interaction zone during habituation (*F*-test, *p* = 0.872; *t*-test assuming equal variance, *p* = 0.0673) or test phase (*F*-test, *p* = 0.0972; *t*-test assuming equal variance, *p* = 0.604), or in **(F)** absolute time spent in the corner zone during habituation (*F*-test, *p* = 0.538; *t*-test assuming equal variance, *p* = 0.221) or test phase (*F*-test, *p* = 0.818; *t*-test assuming equal variance, *p* = 0.486). **(G)** SI ratios were calculated as time spent in the interaction zone when target is present divided by time spent in the interaction zone when target is absent and showed no statistical difference between female and male mice (*F*-test, *p* = 0.9559; *t*-test assuming equal variance, *p* = 0.6000). **(H)** Corner ratios were calculated analogously and inverted such that higher scores would indicate more social avoidance analogous to SI ratios and also showed no statistical difference between female and male mice (*F*-test, *p* = 0.5290; *t*-test assuming equal variance, *p* = 0.4566).

**Figure 2:**
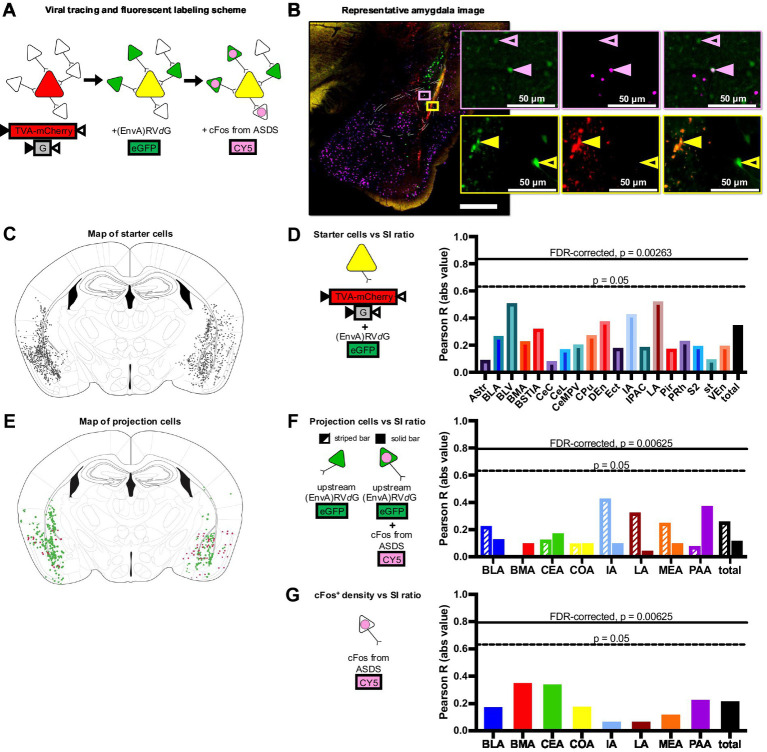
Structural and functional connectivity patterns within the amygdala complex do not mediate individual variability in stress response. **(A)** Viral tracing and fluorescent labeling scheme show colors associated with each additional fluorescent marker (red = TVA-mCherry-G, green = oG-rabies-GFP, magenta = cFos). **(B)** Example triple-channel image of the amygdala region (red = mCherry, green = GFP, magenta = cFos). Top inset (pink box and outline) shows examples of a structural projection neuron (open pink arrow, GFP^+^cFos^−^) and a functional projection neuron (closed pink arrow, GFP^+^cFos^+^). Bottom inset (yellow box and outline) shows examples of a starter neuron (closed yellow arrow, GFP^+^mCherry^+^) and a projection neuron (open yellow arrow GFP^+^mCherry^−^). **(C)** Map of all identified starter cells (GFP^+^mCherry^+^). **(D)** Correlations between starter cell count in each region and SI ratio with FDR-corrected and non-FDR-corrected significance threshold indicated (solid horizontal line, Pearson *R* = 0.835, *p* = 0.00263; dashed horizontal line, Pearson *R* = 0.632, *p* = 0.05; respectively). Starter cell counts did not predict behavioral phenotype in any identified brain region. **(E)** Map of all identified structural (GFP^+^cFos^−^) and functional (stress-activated) projection (GFP^+^cFos^+^) neurons. **(F)** Correlations between structural projection neuron (GFP^+^cFos^−^) count (striped) and functional projection neuron (GFP^+^cFos^+^) count (solid) in each region and SI ratio with FDR-corrected and non-FDR-corrected significance threshold indicated (solid horizontal line, Pearson *R* = 0.749, *p* = 0.0125; dashed horizontal line, Pearson *R* = 0.632, *p* = 0.05; respectively). No significant correlations identified. **(G)** Correlations between overall number of activated cells (cFos^+^) per brain region and SI ratios.

Mice were injected with the helper virus AAV8-CMV-TVA-mCherry-2A-oG (0.5–0.9 μL, Salk Institute California, titer of 1.28 × 10^8^ TU/mL) into the BLA bilaterally (from bregma: AP −1.03, ML ±3.34, DV −4.89, angle 0°) under a combined isoflurane anesthetic (2–5%, SomnoSuite, Kent Scientific) and meloxicam analgesic (5 mg/kg) protocol (Figure 1A). Mice were allowed to recover and fully express the virally delivered mCherry-tagged TVA and G-protein complexes for 3 weeks. GFP-tagged glycoprotein-deleted pseudorabies virus, AAV8-RV*dG*-eGFP (0.5–0.9 μL, Salk Institute California, titer of 2.72 × 10^8^ TU/mL), was then injected into the BLA of each hemisphere (from bregma: AP −1.03 mm, ML ±4.43 mm, DV −5.09 mm, angle 11°) (Figure 1B) and mice were given a week prior to behavioral testing to allow for full recovery and expression of the GFP-tagged glycoprotein-deleted pseudorabies virus. Post-hoc histological confirmation of BLA-centered targeting was performed on all brains (Figure 2C) and only those meeting viral localization were included in further analysis.

### 2.3 Acute social defeat stress

One hour before beginning ASDS, urine was collected from nonaggressive CD1 mice and thoroughly applied (10–25 μL) to the urogenital region of all female C57 mice as previously described (Harris et al., 2018) to increase the propensity of CD1 aggression upon female mice (Mugford and Nowell, 1970; Chamero et al., 2007). Each male C57 was placed into the home cage of a CD1 aggressor for 2 min. This was repeated three times sequentially with three different CD1 aggressors, with no rest periods. Experimental female mice were maintained in group housing in the experimental room during the CD1-on-male C57 aggression and were then each individually exposed to acute stress in an identical paradigm as to which the males were exposed (Figure 1C). As previously reported (Grossman et al., 2022), all mice were singly housed for the remainder of the hour and tested for SI 60 min following the onset of ASDS. For this experiment, no control C57 mice were included, as the focus of the study was individual variability in behavioral response.

### 2.4 Social interaction test

Precisely 1 h after the onset of the first defeat in ASDS, C57BL/6J mice were placed into an open-field arena (45 cm × 45 cm) with a removable wire-mesh enclosure secured in clear Plexiglas (10 cm W × 6 cm D × 30 cm H) placed against the middle of one of the inner walls of the arena. Mice were allowed to habituate to the arena for 2.5 min. After 2.5 min, a novel non-aggressive CD1 “target” mouse was placed in the wire-mesh enclosure. The C57BL/6J mouse was then allowed to explore for another 2.5 min (Figure 1D). Open-field arenas and wire-mesh enclosures were wiped with a cleaning solution between tests. All tests were conducted during the lights on period (between 13:00 and 19:00) under infrared light conditions. A video-tracking system (Ethovision XT 15, Noldus Information Technology) was used to record the movements of the C57 mouse during the “target absent” and “target present” periods. The total time spent in the “interaction zone,” defined as a 24 cm × 8 cm corridor surrounding the wire-mesh enclosure, and corner zones, 9 cm × 9 cm in the two corners opposite from the interaction zone, was recorded. The SI ratio was calculated by the absolute time spent in the interaction zone (blue, Figure 1D) with the target present divided by the absolute time spent in the interaction zone with the target absent. The corner ratio was calculated analogously, as the absolute time spent in the corner zone (red, Figure 1D) with the target present divided by the absolute time spent in the corner zone with the target absent (Grossman et al., 2022), and was then inverted for enhanced comparability with SI scores (i.e., lower scores predict social avoidance, and higher scores predict social approach).

### 2.5 Tissue collection and processing

Mice were anesthetized with isoflurane, checked by toe pinch, and sacrificed by transcardial perfusion with 4% paraformaldehyde immediately after SI testing. Each perfusion started one-hour post-ASDS to support literature findings of peak cFos protein levels occurring between 60 and 90 min after cellular activation (Zinck et al., 1993; Skar et al., 1994; Li et al., 1997). The 60-min mark was chosen to ensure the capture of activated neurons was most time-locked to the first exposure to the social stressor. Perfusions were performed at a flow rate of 9 mL/min for 10 min, followed by decapitation and submandibular cuts for increased fixation. Brains were post-fixed for 72 h, deskulled, and sectioned at 100 μm using a vibratome (speed 8.5, frequency 9; Leica Biosystems), at which point the left hemisphere was marked with a “nick” for hemisphere-specific analysis of injection accuracy. Sectioned brains were stored in 0.9 M PBS with 0.5% sodium azide until immunohistochemical analysis was performed. Sections were serially stained with rabbit anti-cFos (9F6) primary monoclonal antibody (Cell Signaling, catalog number: 2250S, lot number: 12, 1:2,000 in 0.3% PBST + 5% Normal Goat Serum + 1% BSA + 0.2% Cold Water Fish Skin Gelatin block solution) and Alexa Fluor-conjugated AffiniPure goat anti-rabbit secondary antibody (Alexa Fluor-647, Sigma, code number: 111-605-033, lot number: 157045, 1:500 in 0.3% PBS-T). All sections were incubated in 4′,6-diamidino-2-phenylindole (DAPI) for histological identification.

### 2.6 Imaging and analysis

A Nikon Eclipse Ti2-E Motorized inverted microscope and the Element Acquisition Software were used for all imaging. Initially, all coronal sections along the wide brain were scanned for long-range projections, but upon identification of only local projections, sequential amygdala sections were imaged for analysis. For histological confirmation of injection sites, low resolution (4×) 16-bit images of sections spanning the entire anterior-posterior extent of the amygdala were inspected. Triple or quadruple channel *z*-stacks (stacks of 2 μm thickness spanning a total of 20 μm) were then acquired to image GFP, mCherry, CY5, and sometimes DAPI signal and the EDF function of the Element Software applied to collapse the *z*-stack. For projection analysis of cFos, GFP-tagged Rabies, and mCherry-tagged TVA-G cells (Figure 2A), higher resolution (10×, and 40×) 16-bit images were acquired at the amygdala (Figure 2B). Further analysis was done on two selected sections for each animal, one corresponding to the anterior part of the amygdala complex (AP −1.2 to −1.5 from bregma) and one to the more posterior part (AP −1.8 to −2.1 from bregma). Due to the strength of Cy5, the laser power was tuned as low as possible to detect cFos^+^ cells, to minimize leakage from the mCherry fluorescent reporter. Cross comparison was also performed on mCherry-tagged cells dorsal to the amygdala that were infected along the injection line, but that were expected to be cFos^−^ following social stress and it was confirmed that these cells were not detected in the Cy5 channel. Notably, these cells did not receive GFP-Rabies virus, due to the multi-angle injection approach, and were thus not confounding starter cell populations, allowing for the use of this mild off-target infection as a confirmation of Cy5 filter specificity. The nd2 files acquired were imported in Fiji (Schindelin et al., 2012) and modified for counting and reconstruction analysis. Each split channel was smoothed and a seven pixels BG subtraction applied. Before proceeding to counting, non-rigid free-form registration of the selected 100 μm sections was performed on the GFP channel with the open-source WholeBrain Software (Fürth et al., 2018) in RStudio. Briefly, each image was first matched to a corresponding plate of the Mouse Allen Brain Reference Atlas CCF v3 (Wang et al., 2020). The contour of the brain section was segmented out using the autofluorescence of the down-sampled section itself, and a set of correspondence points that align the selected reference atlas plate with the tissue-section was automatically generated by the “thin-plate splines algorithm.” Landmarks such as ventricles were used to manually adjust, add, or remove correspondent points when necessary. The two sets of reference points (atlas and tissue-section reference points) constitute a 2D final mapping of each brain region along the antero-posterior axis. The extracted warped contours of the amygdala complex were exported from RStudio and overlapped to the original 16-bit images in ImageJ (Schneider et al., 2012) with a custom-made macro (Mahadevia et al., 2021). GFP Rabies^+^ cells were counted after thresholding the resulting images (analyze particle plugin). CY5 cFos^+^ cells were counted after applying a white top hat to the images (MorphoLibJ plugin). For colocalization analysis of starter cells (GFP Rabies^+^ mCherry TVA-G^+^) and stress-activated projection cells (GFP Rabies^+^ CY5 cFos^+^), the image calculator was implemented using the AND function. Method validation was performed with the coloc function. The schematic of projections and starter cells for the left and right hemispheres (Figures 2C,E) were reconstructed in ImageJ (Schneider et al., 2012) by overlapping and realigning in a stack the segmented images for the channel of interest over the representative plate from the Paxinos Mouse Brain Atlas 4th edition (Paxinos and Franklin, 2012) in Adobe Illustrator V for final color modifications. All statistical analyses including correlations and FDR corrections were performed in excel using the built-in statistical functions.

## 3 Results

### 3.1 Behavioral results

Following adequate recovery time from stereotactic viral injections (Figures 1A,B), mice were exposed to our previously developed ASDS paradigm (Grossman et al., 2022), which consists of three consecutive two-minute bouts of social stress (Figure 1C) and was here extended to include female mice. After 1 h of rest, the experimental mice underwent an SI test as a behavioral readout of individual variability in behavioral response to stress, measured as time spent in the interaction zone, near a novel “target” mouse of the same breed as the aggressor, and the time spent in the corner zones (Figure 1D). Given the explicit readout of individual variability in behavioral response to stress, all mice underwent the social stress paradigm and unstressed controls were not included. ASDS elicited a spectrum of behavioral responses ranging from social avoidance to social approach in both female (*n* = 5) and male (*n* = 5) mice (Figures 1E–H). Female and male mice showed no statistically significant difference in time spent in the interaction zone (repeated measures ANOVA: F vs. M, *p* = 0.135; phase, *p* = 0.0863; interaction, *p* = 0.522) (Figure 1E), corresponding to SI ratio (*t*-test: *p* = 0.6000) (Figure 1G), or time spent in the corner zone (repeated measures ANOVA: F vs. M, *p* = 0.198; phase, *p* = 0.0456; interaction, *p* = 0.899) (Figure 1F), corresponding to inverse corner ratios (*t*-test: *p* = 0.4566) (Figure 1H). These metrics provide a justification for concatenating female and male data for a total sample size of 10 animals that could be used for analyses exploring neural correlates of individual variability in behavioral stress responses.

### 3.2 Cellular results

Post-hoc histological confirmation of the injection site was performed on all brains and overlaid with DAPI for regional identification of the amygdala. All 10 animals demonstrated injection endpoints of both the mCherry-tagged TVA and G-protein containing viral complex and the GFP-tagged pseudotyped G-deleted rabies virus (Figure 2C) within the bilateral amygdala complex. After thorough optimization of immunohistochemical conditions, consistent cFos fluorescence was observed in all 10 animal samples. Since all animals met viral localization criteria satisfactorily (Grossman et al., 2022), no subjects were excluded from analysis. Two amygdala sections from each brain were imaged in detail, one from the more anterior portion of the amygdala complex (AP −1.2 to −1.5 from bregma) and one from the more posterior end of the amygdala complex (AP −1.8 to −2.1 from bregma) (example in Figure 2B). Since there were no identifiable differences in hemispheric projections, projections from both hemispheres were analyzed in all animals. Starter cells (GFP^+^mCherry^+^) were identified across the amygdala complex as well as surrounding cortical regions in a total of 19 subregions, and starter cell location and total number was not predictive of the behavioral phenotype (Figure 2D). Eight of the 13 amygdala subregions also contained projection cells (GFP^+^mCherry^−^), so further analyses were restricted to these eight subregions. By controlling for the individual variability in the infection rate using total starter cell number per animal (GFP^+^mCherry^+^), structural (not responsive to stress, GFP^+^cFos^−^) and, functional (stress-activate, GFP^+^cFos^+^) projection cell counts, as well as alternately activated cFos expression (GFP-cFos+) compared to behavioral response were compared across these eight amygdala-associated subregions (Figures 2E–G) No correlations between structural or functional projection cell number and SI scores in any of the eight amygdala subregions reached FDR-corrected significance threshold, though we propose this methodological approach for future investigation.

## 4 Discussion

Here, we present a proof-of-concept study that integrates two advanced methodologies to examine how local brain connectivity might predict individual behavioral variability in response to social stress. By combining the retrograde transsynaptic rabies system and immediate early gene immunohistochemistry, we show how correlations can be made between this structural and functional connectivity with behavioral responses, using an acute social stressor that was previously validated to lead to high degrees of individual variability in social approach/avoidance at 1 h post-stress initiation. Crucially, our methods provide ability to examine both structural and functional connectivity within the same animal, allowing for a more accurate comparative analysis.

The methodology described present some advantages for investigating local connectivity. First, the transsynaptic rabies system provides a higher level of specificity than generic AAV tracers. The TVA-mCherry-oG viral complex capitalizes on the lack of expression of TVA in mammalian neurons to target future introduction of RV*dG* into specific cells. Glycoprotein-deleted pseudorabies (RV*dG*) is typically pseudotyped with envelope protein from avian ASLV type A (EnvA), which uses the TVA receptor for entry into the cell. Thus, RV*dG* can only enter cells that first were infected with the AAV8 helper virus carrying both the glycoprotein and the TVA receptor, the latter now expressed on their surface (Callaway and Luo, 2015). With the subsequent injection of eGFP-tagged RV*dG*, the cells infected with both viral complexes fluoresce yellow (red + green) and due to co-expression of the glycoprotein needed for RV*dG* transsynaptic transfer, can jump retrograde to label upstream targets green. Importantly, transsynaptic transfer can only occur once, upstream neurons lacking the necessary glycoprotein. It should be noted that the AAV8 helper virus has the potential to perform both anterograde and retrograde transport (Castle et al., 2014), indicating that fluorescence of the mCherry tag in starter cells could represent anterograde or retrograde uptake of TVA-mCherry-oG. However, rabies virus particles only jump retrograde in the central nervous system, infecting neurons transsynaptically through axon terminals and spreading upstream through the synapse to neurons presynaptic to the starter cells (Wickersham et al., 2007). Importantly, anterograde rabies viral particle jump has been observed in peripheral neurons (Xu et al., 2020; Potratz et al., 2020; Bauer et al., 2014), in somatosensory neurons transmitting signal directly to the spinal cord (Zampieri et al., 2014), or when coated with the envelope glycoprotein of vesicular stomatitis virus to co-op intrinsic stomatitis anterograde properties (Wickersham, 2013; Beier et al., 2011). Therefore, in our experiment GFP^+^mCherry^−^ projection cells represent monosynaptic retrograde transport of RV*dG* from GFP^+^mCherry^+^ starter cells, despite antero- or retrograde transmission of the AAV8 helper. Moreover, the dual angles employed for the two sequential injections ensure high specificity of injection localization in a deep structure. Bilateral injections were chosen following pilot experiments of unilateral injections showing no long-range or contralateral labeling protection neurons. While long-range monosynaptic connections to the amygdala are well-described (Anderson and Dumitriu, 2020; Anderson et al., 2021), our methods only resulted in short-range, ipsilateral projection tracing. We acknowledge that likely individual stress responses result from both long- and short-range connections to the amygdala, but here we highlight a less thoroughly understood mechanism of short-range projections as they mediate the social stress response. Notably, the helper virus used in our experiment, AAV8-CMV-TVA-mCherry-2A-oG, has only been used to trace short-range projections to the best of our knowledge (Ciabatti et al., 2017). Thus, the combination of utilizing a short-range helper virus serotype, low viral titers, and dual angled injections allowed us to illuminate local projections that had thus far been hidden by viral overload using other viral combinations. That being said, we implement a three-week expression period following injection of the helper virus but only a one-week expression period following pseudorabies injection. This expression period was limited to 1 week due to pseudorabies’ generally high cytotoxicity levels which can trigger cell death in infected cells after 1–2 weeks (Qiu et al., 2022). However, it is possible that the relatively short expression period following the second injection limited the chance for long range presynaptic cells to become infected.

Our proposed method as it stands, has several limitations, the first being the lack of specificity of the BLA injection site. While the proof-of-concept results we present provide a basis of experimental methods for future investigation, it is true that correlative quantification of BLA projections with starter cells outside of the BLA cannot rule out the contribution of projections to non-BLA amygdala subregions to the social stress response. We encourage the implementation of our combined injection and behavior methodology to decipher more mechanistic links between specific subregions with perhaps even lower virus titer and/or more strict exclusion criteria for animals with a wide spread of starter cells. The exclusivity of short-range connectivity in this study serves as both a limitation and a strength given the ability to now pinpoint local circuits, particularly in the amygdala complex, known to be important but whose direct roles on behavior remain largely elusive. We believe this approach may lead to future dissection of local and micro circuits in other brain regions, yet to be discovered.

For enhanced translatability, our behavioral design focuses on a spectrum of SI ratios that more effectively approximates the range of behavioral phenotypes seen in the human clinical setting (Cuthbert, 2015; Cuthbert, 2020) than the commonly employed dichotomous classification of “social avoidance” versus “social approach.” Current diagnostic methods used in psychiatry often group symptoms into systematic categories within the DSM rather than acknowledging a wide spectrum of phenotypes in which a symptom can present. Thus, evaluating behavioral responses on a spectrum, rather than grouping mice into categories, better encompasses and mirrors the wide range of symptomatic behaviors presented in the human clinic.

Despite the robust behavioral classification utilized in this study, it is important to note that some animals spent less time in the interaction zone even during the habituation stage of the assay. It is possible that mice spent less habituation time in the interaction zone due to increased anxiety that is not related to the presence of a CD1 aggressor, given that some animals spent less time in the interaction zone during habituation. A future way to delineate that less time spent in the interaction zone and higher corner ratios are strictly due to social avoidance could be to test a cohort of unstressed control mice in this ASDS paradigm. Additionally, a positive control group of mice that have undergone a non-social stressor may help further parse out the behavioral components of social avoidance from unrelated anxiety. These experiments should be performed in future behavioral research, however it was beyond the scope of our proof-of-concept methodology. Future experiments should also use a greater number of both male and female mice to uncover sex-specific differences that were likely masked due to the limited n in this study.

As presented, the methodology used here serves as a powerful pilot towards elucidating how connectivity and activity within a localized brain region can be indicative of trait vulnerability to stress. Through the approach of preclinical classification of a social stress response, associating behavioral phenotypes with neuronal correlates, and ultimately identifying target brain regions and circuits that mediate the stress response, we begin to identify potential clinical targets for preventative screening for neuropsychiatric disorders as well as further individualized treatment.

## Data Availability

The raw data supporting the conclusions of this article will be made available by the authors, without undue reservation.
